# Herb-Drug Interaction: Application of a UPLC-MS/MS Method to Determine the Effect of *Polygonum capitatum* Extract on the Tissue Distribution and Excretion of Levofloxacin in Rats

**DOI:** 10.1155/2020/2178656

**Published:** 2020-08-18

**Authors:** Li Yuan, Hao Chen, Xue Ma, Jie Pan, Zi-Peng Gong, Si-Ying Chen, Yue-Ting Li, Ai-Min Wang, Lin Zheng, Yong Huang

**Affiliations:** ^1^State Key Laboratory of Functions and Applications of Medicinal Plants, Guizhou Provincial Key Laboratory of Pharmaceutics, Guizhou Medical University, Guiyang 550004, China; ^2^School of Pharmacy, Guizhou Medical University, Guiyang 550004, China; ^3^Guizhou Provincial Engineering Research Center for the Development and Application of Ethnic Medicine and TCM, Guizhou Medical University, Guiyang 550004, China

## Abstract

*Polygonum capitatum* has unique curative effects on the urinary system. In fact, many *Polygonum capitatum*-based preparations are currently used in the clinic. In China, the combination of levofloxacin (LVFX) with a Chinese herbal preparation derived from *Polygonum capitatum* has been used for the clinical treatment of urinary system diseases, which can improve the curative effects and reduce the side effects of LVFX. However, the herb-drug interaction (HDI) between these drugs has not been reported and the effect of *Polygonum capitatum* on the in vivo process of LVFX is unclear. In this article, a sensitive ultraperformance liquid chromatography-tandem mass spectroscopy (UPLC-MS/MS) method was developed to evaluate the effects of the combined application of LVFX and the *Polygonum capitatum* extract on tissue distribution and excretion. Thereafter, the method was validated for selectivity, accuracy, precision, linearity, lower limit of quantification (LLOQ), dilution integrity, recovery, and matrix effect. Based on tissue distribution, LVFX could diffuse into all of the tested tissues, with significant differences in the content of each tissue between the coadministration group and single administration group. At 48 h after the combination was orally administered, the urinary cumulative excretion of LVFX decreased from 20.69% to 11.84% while its fecal cumulative excretion decreased from 26.08% to 13.28%. Our results suggest that a drug interaction exists between the two drugs in the process of distribution and excretion. This study provides important experimental evidence for further studies on the clinical efficacy and mechanism of the *Polygonum capitatum* extract and LVFX.

## 1. Introduction

For thousands of years, traditional Chinese medicine has been widely used in China to treat diseases. With the development of modern medicine, more and more people are trying to combine herbs and western medicines clinically to explore new medication options [1–3]. A reasonable herb-drug interaction (HDI) can enhance efficacy and reduce adverse reactions [4]. For example, the combination of *Eugenia jambolana* extract and sitagliptin caused significant improvement in the comorbidities associated with diabetes mellitus compared to sitagliptin treatment alone [5]. *Pittosporum angustifolium* Lodd. extracts also potentiated the activity of conventional antibiotics, without significantly affecting the toxicity of the combination [6]. However, the composition of Chinese herbal medicine is complex, and there is little information about the pharmacokinetics of many phytochemicals present in herbal medicine. When used with western medicine especially, coadministration of herbs and conventional medicines will lead to increased potential risks of herbs interacting with the medicine; for example, combining ginseng with warfarin could increase the risk of bleeding in patients on chronic warfarin therapy [7], which represents huge challenges in terms of safety of the medication. Therefore, it is essential to evaluate the safety and effectiveness of HDI and drugs by studying the pharmacokinetics of the drug *in vivo* when combining herbs with western medicine.

Levofloxacin (LVFX) is used to treat numerous bacterial infections belonging to the third-generation fluoroquinolone antibiotic family [8]. In fact, it is safe and effective and is widely employed to treat diseases such as community-acquired pneumonia, acute bacterial sinusitis, complicated urinary tract infections, and acute pyelonephritis [9–11]. Because of the widespread use of this drug, its incidence of adverse reactions has increased. These adverse effects including abdominal discomfort, diarrhea, vomiting, headache, skin rash, pruritus, and headache are commonly identified during clinical treatment [12–14]. Thus, in clinics in China, many doctors opt to prescribe a combination of LVFX and a Chinese patent drug to enhance its efficacy and reduce its adverse effects. *Polygonum capitatum* is derived from *Polygonum capitatum* Buch.-Ham. ex D.Don and is a well-known Miao medicine that is widely used to treat different urinary system diseases [15]. Some drugs that are derived from *Polygonum capitatum* are presently used in the clinic such as Relinqing® granule and Milins® capsules [16]. Relinqing® granule, a unilateral preparation derived from *Polygonum capitatum*, is the best-selling Chinese patent drug for the treatment of urinary diseases and has been included in Chinese pharmacopoeia 2015 [17]. For patients with urinary system infection, Relinqing® granule from *Polygonum capitatum* is usually combined with LVFX. Several clinical prospective studies reported that the combination of a Chinese herbal preparation derived from *Polygonum capitatum* and LVFX is more effective and can thus enhance their efficacy to treat patients with urinary system infection, shorten the course of treatment, reduce the adverse effects of antibiotics, and exhibit a significant synergistic effect [18–20]. However, published studies have mainly focused on the clinical curative effect after the two drugs are combined. To our knowledge, some information regarding the tissue distribution and excretion of LVFX combined with *Polygonum capitatum* is lacking. Furthermore, the interactions and mechanism of the active ingredients in HDI are unclear.

In vivo distribution studies are crucial in drug research as they can be performed to demonstrate the pharmacokinetic features of a drug [21, 22]. In particular, investigating drug distribution throughout the whole body is critical as it provides insights into the accumulation and metabolism of drugs in a particular tissue [23]. In the present work, we examined the tissue distribution and excretion behavior of the single administration of LVFX or its combination with *Polygonum capitatum*. In addition, we established an LC–MS/MS method to simultaneously determine LVFX in tissue homogenate, urine, and feces after the oral administration of LVFX and *Polygonum capitatum*. By comparing the distribution and excretion results, we opted to examine the differences achieved under the two modes of administration to understand the changes in LVFX in rats. Therefore, the potential HDI in rats was examined via tissue distribution and excretion studies to comprehensively reveal its safety, predict its mechanism, and clarify its clinical applicability.

## 2. Materials and Methods

### 2.1. Chemicals and Reagents

*Polygonum capitatum* was extracted with water according to the Chinese pharmacopeia [17]. LVFX (purity: 97.3%) and puerarin (purity: 95.4%) standards were obtained from the National Institutes for Food and Drug Control (Beijing, China). Methanol, formic acid, and acetonitrile (HPLC-grade) were purchased from Merck Co. (Darmstadt, Germany). Distilled water was obtained from Watsons Group Co. Ltd. (Hong Kong, PR China). All other chemicals were of analytical grade.

### 2.2. Instrumentation and Conditions

An ACQUITY I-Class UPLC system with a conditional autosampler and an Acquity I-Class UPLC BEH C_18_ Column (2.1 mm × 100 mm, internal diameter 1.7 *μ*m) was used for the analyses. The system was also equipped with a Waters VanGuard BEH C_18_ (2.1 mm × 5 mm, 1.7 *μ*m) column. The column and autosampler were maintained at 40°C and 25°C, respectively. The injection volume was 1 *μ*L. The gradient mobile phase system consisting of 0.1% formic acid in acetonitrile (B) and 0.1% aqueous formic acid (A) was applied at a flow rate of 0.3 mL/min and run time of 4.0 min. The following gradient elution program was carried out for chromatographic separation: 0.0–0.3 min (90% A), 0.3–1.0 min (90%–85% A), 1.0–2.0 min (85%–90% A), 2.0–3.0 min (90%–90% A), and 3.0–4.0 min (90%–10% A).

Mass spectrometric detection was performed with an XEVO TQS Triple-Quadrupole Tandem Mass Spectrometer (Waters Corp, Milford, MA, USA) equipped with an electrospray ionization (ESI) source. The mass spectrometer parameters were capillary voltage, 3.0 kV; capillary ionization voltage, 3 kV; ion source temperature, 120°C; spray gas and backflush gas, N_2_; desolvation gas flow rate, 650 L·h^−1^; desolvation gas temperature, 350°C. Multiple reaction monitoring (MRM) mode was used for quantification. The optimal parameters for the analyte and internal standard (IS, puerarin) in the MRM mode are listed in Table 1. All data were obtained using MasslynxTM V 4.1 software and processed using the QuanlynaTM V4.1 (Waters Corp., Millford, MA, USA) workstation.

### 2.3. Animal

Sprague–Dawley rats (230 ± 20 g) were supplied by Changsha Tianqin Biotechnology Co. Ltd. (Changsha, China; certificate No. SCXK (Xiang) 2014-0010). All studies were approved by the Animal Ethics Committee at Guizhou Medical University and conducted in accordance with the guidelines of the Committee on the Care and Use of Laboratory Animals in China.

### 2.4. Standard and Sample Preparation

#### 2.4.1. Stock Solutions, Calibration Standards, and Quality Control (QC) Sample Preparation

The stock solutions of LVFX were separately weighed and dissolved in methanol to obtain the final concentration of 1.002 mg/mL. An appropriate amount of puerarin was dissolved in methanol and diluted to obtain the IS solution (20 ng/mL). The stock solutions of LVFX were successively diluted to the following concentrations to generate the calibration curves: 5.01–15,030.00 ng/mL, 5.01–10,020.00 ng/mL, and 5.01–1002.00 ng/mL. Quality control (QC) samples containing 10.02, 100.20, and 5010.00 ng/mL of LVFX were prepared for the tissue distribution study, 10.02, 200.40, and 4008.00 ng/mL of LVFX for the urinary excretion study, and 10.02, 20.04, and 200.40 ng/mL of LVFX for the fecal excretion study. All stock and working solutions were stored at 4°C and brought to room temperature before use.

#### 2.4.2. Sample Preparation

The tissues were cut on ice and mixed evenly. Each weighed tissue was homogenized in physiological saline (1 : 4, w/v) after thawing. The corresponding tissue homogenate with no drug was used as the blank homogenate.

One milliliter of tissue homogenate and the bladder homogenates were centrifuged (5000 rpm, 4°C, 8 min). Thereafter, 50 *μ*L of the IS solution (puerarin, 20 ng/mL in methanol) and 50 *μ*L of 2% formic acid were added to 100 *μ*L of the rat tissue homogenate. For protein precipitation, 400 *μ*L of methanol was added to the protein sample, vortexed for 1 min, sonicated for 10 min, and centrifuged at 12,000 rpm for 10 min at 4°C. The supernatant was collected in a centrifuge tube and dried at 37°C with N_2_. Finally, 150 *μ*L of the sample was dissolved in 50% methanol, vortexed for 1 min, sonicated for 10 min, and centrifuged at 4°C for 10 min at 12,000 rpm. The supernatant was then analyzed by UPLC-MS/MS.

Feces were crushed with a grinder. Thereafter, 0.5 g of the feces was homogenized with physiological saline (1 : 8, w/v), vortexed for 2 min, sonicated for 10 min, and centrifuged at 5000 rpm for 10 min at 4°C. The supernatant was then collected in a centrifuge tube.

A 100 *μ*L volume of rat urine or fecal homogenate was diluted 100-fold with the corresponding blank matrix. Thereafter, 50 *μ*L of the IS solution (puerarin, 20 ng/mL in methanol) and 50 *μ*L of 2% formic acid were added to 100 *μ*L of the diluted rat urine or fecal homogenate. For protein precipitation, 800 *μ*L of methanol was added to the protein sample, vortexed for 1 min, sonicated for 10 min, and centrifuged at 12,000 rpm for 10 min at 4°C. The supernatant was then collected in a centrifuge tube and used in the UPLC-MS/MS analysis.

### 2.5. Method Validation

#### 2.5.1. Selectivity

The specificity of the method was evaluated by analyzing a blank rat liver tissue homogenate, adding LVFX and IS to this blank, and collecting the tissue homogenate at 30 min after oral administration.

#### 2.5.2. Linearity and Lower Limit of Quantification

As described in Section 2.4, a standard curve was established to evaluate the linearity by plotting the relationship between the peak area ratio (*y*) of the analyte and IS and the standard concentration (*x*) of the analyte using 1/*x*^2^ weighted least-squares linear regression. The LLOQ should satisfy the analytical requirement of a signal-to-noise ratio (S/N) of ∼10.

#### 2.5.3. Precision and Accuracy

The precision and accuracy of the test were determined by analyzing the quality control samples of the five replicates at three concentration levels (low, medium, and high) on the same day (intraday) and three consecutive days (interday).

#### 2.5.4. Extraction Recovery and Matrix Effect

The extraction recovery of the analyte was evaluated by comparing the peak area ratio of the low concentration, medium concentration, and high concentration of the pretreated QC samples to the peak area ratio of the supernatant containing the same concentration of the standard solution. The matrix effect was evaluated by comparing the peak area of the analyte in the spiked sample postextraction, with the peak area of the analyte dissolved in the same concentration of methanol. Five replicate analyses were performed on the QC samples.

#### 2.5.5. Stability

The stability of the analytes was determined using low, medium, and high concentrations (*n* = 5 for each concentration level) under different conditions: 6 h at room temperature, three freeze (−20°C) and thaw (room temperature) cycles, and storage at 4°C for 12 h.

#### 2.5.6. Dilution Integrity

The dilution integrity of the test was determined by analyzing the high-concentration samples in the ultralinear range (urine: 1,002.00 *μ*g/mL; feces: 100.20 *μ*g/mL) following 100-fold dilution with the blank rat matrix. A result within ±15% should be achieved to satisfy the deviation between the measured result and the labeled amount.

### 2.6. Tissue Distribution and Excretion Study

For the tissue distribution study, Sprague–Dawley rats were randomly divided into two groups, with 24 rats in each group. Group one was administered 42 mg·kg^−1^ LVFX while group two was administered 1.86 g·kg^−1^*Polygonum capitatum* extract and 42 mg·kg^−1^ LVFX (dose selection: converted according to the clinical dose). The heart, liver, spleen, lung, kidney, stomach, intestine, and bladder were, respectively, collected at 15, 30, 120, and 360 min after oral administration (*n* = 6, each point). Each tissue was washed with normal physiological saline, dried with filter paper, weighed, and stored at −80°C until analysis.

For the urinary and fecal study, Sprague–Dawley rats were randomly divided into two groups (6 rats/group). Group one was administered 42 mg·kg^−1^ LVFX while group two was administered 1.86 g·kg^−1^*Polygonum capitatum* extract and 42 mg·kg^−1^ LVFX. Rats were housed in stainless-steel metabolic cages and granted free access to food. Urine was collected at different time points (0–2, 2–4, 4–8, 8–12, 12–24, 24–36, and 36–48 h) after oral administration. Fecal samples were collected at different time points (0–12, 12–24, 24–36, and 36–48 h) after oral administration. After urine volume and fecal dry weight were measured in each collection period, the biological samples were stored at −80°C.

### 2.7. Statistical Analysis

Data are presented as mean ± standard deviation (SD). Statistical analysis was performed using the statistical software package, Statistical Product and Service Solutions (SPSS 11.5, SPSS Inc., Chicago, IL, USA). Factorial analysis of variance was used for comparison between groups. A *P* value <0.05 was considered to indicate statistical significance, while *P* values <0.01 indicated very significant difference.

## 3. Results

### 3.1. Method Validation

#### 3.1.1. Selectivity

**Figure 1:**
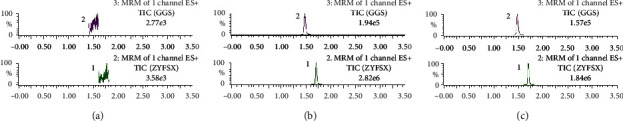
Typical chromatograms. (a) Blank tissue homogenate; (b) blank tissue homogenate spiked with LVFX and IS; (c) rat tissue homogenate collected at 30 min after oral administration of LVFX. (1) LVFX; (2) puerarin.

Figure 1 shows the chromatograms of a blank tissue homogenate sample, a tissue homogenate sample with LVFX and IS, and a chromatogram obtained from a sample of rat tissue homogenate 30 min after the addition of the LVFX and IS. Based on the retention time, the endogenous substances in the tissue did not interfere with the determination of the analyzed components, indicating the good specificity of the experimental conditions.

#### 3.1.2. Linearity and LLOQ

As described in Section 2.4, a standard curve was established to examine linearity by plotting the relationship between the peak area ratio (*y*) of the analyte and IS and the standard concentration (*x*) of the analyte using 1/*x*^2^ weighted least-squares linear regression; the LLOQ should satisfy the analytical requirement of a signal-to-noise ratio (S/N) of ∼10.  Table 2 shows the typical calibration curve equations, linear ranges, correlation coefficients, and LLOQ of LVFX. All calibration curves showed good linearities (*R*^2^ > 0.995) within the test ranges.

#### 3.1.3. Precision and Accuracy

The precision and accuracy of LVFX are shown in Table 3. The intra- and interday precision and accuracy of LVFX were assessed by analyzing the quality control (QC) samples at three concentrations in five duplicates. The relative standard deviations of the intraday and interday measurements were less than 15%. These data indicate that the values are within the acceptable range and that the method used is accurate and precise.

#### 3.1.4. Extraction Recovery and Matrix Effects

The mean extraction recovery and the matrix effect of LVFX are shown in Table 4. Based on our findings, the developed method is acceptable and accurate for analyzing LVFX in complex matrices.

#### 3.1.5. Stability

Table 5 shows the stability data of LVFX. The stability of the analytes was determined using low, medium, and high concentrations (*n* = 5) under different conditions: 6 h at room temperature, three freeze-thaw cycles from −20°C to 20°C, and storage at 4°C for 12 h. Based on our findings, the analytes were stable under routine laboratory conditions.

#### 3.1.6. Dilution Integrity

The dilution integrity of LVFX in urine and fecal are shown in Table 6. The diluted samples were analyzed using a calibration curve to derive their integrities, which were within the acceptable limit of ±15%. This finding demonstrates that the developed method could be applied for higher concentrations that exceed the linear ranges during quantitative analysis.

### 3.2. Tissue Distribution and Excretion Study

**Figure 2:**
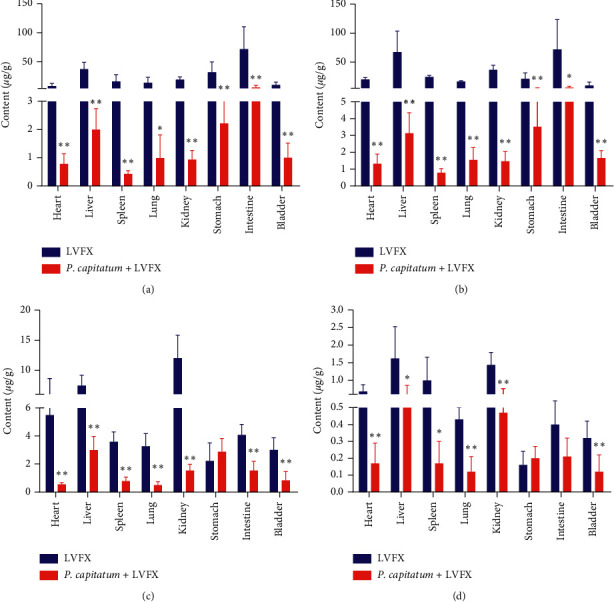
The content of LVFX in rat tissue homogenate of single and coadministration group at four different time points (mean ± SD). Compared with single group: ^*∗*^*P* < 0.05, ^*∗∗*^*P* < 0.01. (a) 0.25 h, (b) 0.5 h, (c) 2 h, and (d) 6 h.

The content of LVFX in the heart, liver, spleen, lung, kidney, stomach, intestine, and bladder is shown in Figure 2, respectively. Compared to the single administration group, the tissue content of the group administered the combination was significantly decreased (*P* < 0.01). Further, when different administration methods were employed, a significant difference was found in the concentration of LVFX in tissues with time (*P* < 0.01). As the data were statistically significant, the concentration of LVFX in tissue could be affected by the drug delivery mode and interaction time. From 30 min to 2 h, a prolongation in the residence time of LVFX in the liver and stomach of rats occurred relative to that of LVFX alone. Such findings demonstrate that elimination in the liver and stomach was reduced after combination treatment.

**Figure 3:**
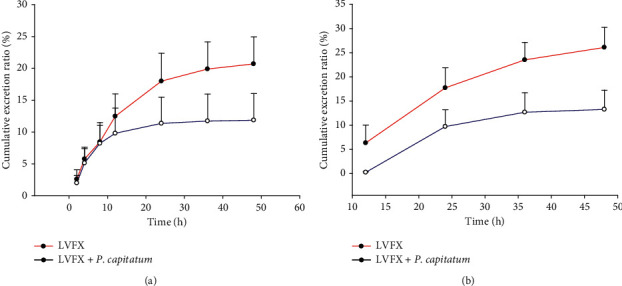
Urinary and fecal cumulative excretion ratio of LVFX after oral administration to rats (*n* = 6). (a) Urinary excretion. (b) Fecal excretion.

Figure 3 showed the excretion of LVFX in urine and feces. At 48 h after the oral administration of a single dose of LVFX (42 mg/kg), and the combination of the *Polygonum capitatum* extract (1.86 g/kg) plus LVFX (42 mg/kg) and the urinary cumulative excretion of LVFX was 20.69% and 11.84%, while its fecal cumulative excretion was 26.08% and 13.28%, respectively.

## 4. Discussion

In the present study, we established a rapid, simple, and sensitive UPLC-MS/MS method. Thereafter, we opted to apply this method to determine the tissue distribution and excretion changes when LVFX was combined with *Polygonum capitatum*. Previously, our research group showed that Relinqing® granule, a unilateral preparation derived from *Polygonum capitatum*, could significantly change the main pharmacokinetic parameters of ciprofloxacin in rats [24], with a decrease in AUC_(0⟶∞)_ and *C*_max_ by 50.0% and 29.3%, respectively, compared to single treatment. LVFX is a safe and effective antibiotic for the treatment of genitourinary infection. In clinics in China, the combination of LVFX and some Chinese herbal preparation derived from *Polygonum capitatum* is widely used to treat genitourinary diseases, as such combination can enhance its efficacy and reduce its adverse effects [18, 20]. Based on these findings, we examined the tissue distribution and the urine and fecal excretion of LVFX alone and combined with the *Polygonum capitatum* extract. Our results aligned with prior findings, as the content of LVFX in vivo significantly decreased after treatment with the combination of the two drugs.

After drugs enter the blood circulation, they are distributed to the tissues of the body and blood. Understanding the characteristics of the tissue distribution of a drug will enable the identification of its target organs and the prediction of its pharmacological effect, which are significant for expanding its clinical use [25, 26]. In this experiment, we investigated the distribution of LVFX in rats for 0.25, 0.5, 2, and 6 h using two drug delivery modes including the absorption phase, equilibrium phase, and elimination phase, which were selected according to the preliminary experimental results. The tissue distribution of LVFX is shown in Figure 2. The drug could be detected in 15 min within the single administration group and the combination group. Thus, LVFX was demonstrated to be rapidly and widely distributed in each tissue under the two different drug delivery modes. The content of tissues in the combined treatment group was significantly reduced relative to that of the single-treated group (*P* < 0.01). *Polygonum capitatum* may inhibit the entrance of LVFX into blood, thereby directly affecting its distribution rate in the tissue.

Generally, multiple mechanisms may be responsible for the HDI of a specific drug. Traditional Chinese medicine mainly causes pharmacokinetic interactions by inhibiting or inducing drug-metabolizing enzymes and transporters, which play a decisive role in the absorption, distribution, metabolism, and excretion of drugs [27]. The cytochrome P450 (CYP450) system and the efflux drug transporter, P-glycoprotein (P-gp), play an indispensable role in most HDIs, and more than half of oral Chinese medicine may interact with the CYP system [28]. According to the literature, the chemical constituents of the *Polygonum capitatum* extract mainly include flavonoids and phenolic acids [29, 30]. Flavonoids can regulate efflux transporters such as p-gp, MRPs, and BCRP [31]. Previously, LVFX, ciprofloxacin, and other quinolones were demonstrated to be the substrates of P-gp [32] and our research group revealed that *Polygonum capitatum* could induce CYP2C9 and CYP3A4 [15]. Thus, after the two drugs were combined, the tissue distribution behavior of LVFX in vivo was altered. The interaction between the two drugs may be related to the metabolic enzymes and transporters in vivo. Therefore, we will carry out a future study on the metabolic enzymes and transporters to determine whether the absorption and distribution of LVFX was altered by the involvement of efflux transporters and metabolic enzymes.

LVFX excretion in urine and feces is shown in Figure 3. When the *Polygonum capitatum* extract and LVFX were combined, their cumulative excretion rate in urine and feces was significantly different from that of LVFX alone (*P* < 0.01). After the *Polygonum capitatum* extract and LVFX were orally administered, the urinary cumulative excretion of LVFX decreased from 20.69 ± 4.26% to 11.84 ± 4.21%, thereby depicting the absorption of the drug followed by its transportation to tissues and organs during blood circulation. According to the results of tissue distribution, drug content of the kidney was significantly reduced after the two drugs were combined, thereby causing a decrease in the excretion of LVFX in urine. Current reports showed that Relinqing® granule from *Polygonum capitatum* is usually combined with LVFX to enhance their efficacy to treat patients with urinary system infection. At present, our current studies at the pharmacokinetic level may not be able to explain this problem strongly. However, drug interactions can be divided into pharmacokinetic interactions and pharmacodynamic interactions. At the pharmacodynamic level, how drugs cause the body to function, the mechanism of action is comprehensive and complex. We speculate whether it is because of the synergistic inhibitory effect of the two drugs on the pathogenic bacteria [33, 34], such as reducing the ability of bacteria to adhere to the urothelial cells, speeding the passage of pathogenic bacteria out of the body, results in improved efficacy, but these speculations need to be confirmed by further experiments. In addition, the fecal cumulative excretion decreased from 26.08 ± 4.16% to 13.28 ± 3.98% at 48 h after oral administration. These results suggest that the *Polygonum capitatum* extract has an effect on the excretion of LVFX in urine and feces. However, a further study is required to elucidate the causes and effects of the emergence of these phenomena.

## 5. Conclusions

Herein, we developed an LC–MS/MS method to determine LVFX in rat biological samples after the combined oral administration of the *Polygonum capitatum* extract and LVFX. Thereafter, we successfully applied this method to a tissue distribution and excretion study in rats. To our knowledge, this is the first study to evaluate the tissue distribution and excretion of *Polygonum capitatum* extract and LVFX when coadministered to rats. Based on our findings, the content of LVFX in tissues and excretion samples was significantly reduced when combined with the *Polygonum capitatum* extract. Moreover, we demonstrated the herb-drug interactions of this drug combination. Nonetheless, a further research on the in vivo HDI between the *Polygonum capitatum* extract and LVFX should be conducted to provide a substantial foundation for investigating the suitability of this combination for clinical applications.

## Figures and Tables

**Table 1:** Mass spectrum parameters of the analyte and puerarin (IS).

Component	Mass spectrum parameters				
	Parent (*m*/*z*)	Daughter (*m*/*z*)	Cone (V)	Collision (V)	ESI
IS	417.00	267.00	40	30	+
LVFX	362.20	261.10	20	10	+

**Table 2:** Calibration curves, linear ranges, correlation coefficients, and LLOQ of LVFX in rat tissues and urine and fecal samples.

Biosamples	Calibration curves	*R* ^2^	Linear ranges (ng/mL)	LLOQ (ng/mL)
Heart	*y* = 0.1582*x* + 2.651	0.9966	5.01–15,030.00	5.01
Liver	*y* = 0.1127*x* + 4.3228	0.9951	5.01–15,030.00	5.01
Spleen	*y* = 0.1837*x* + 3.1029	0.9967	5.01–15,030.00	5.01
Lung	*y* = 0.1418*x* + 2.1413	0.9953	5.01–15,030.00	5.01
Kidney	*y* = 0.1785*x* + 2.8676	0.9961	5.01–15,030.00	5.01
Stomach	*y* = 0.2974*x* + 4.7142	0.9957	5.01–15,030.00	5.01
Intestines	*y* = 0.1583*x* + 2.6446	0.9967	5.01–15,030.00	5.01
Bladder	*y* = 0.1432*x* + 2.2233	0.9954	5.01–15,030.00	5.01
Urine	*y* = 0.0653*x* + 0.4665	0.9998	5.01–10,020.00	5.01
Feces	*y* = 0.0981 + 1.5074	0.9996	5.01–1002.00	5.01

**Table 3:** Precision and accuracy of LVFX in rat liver tissues, urine, and feces (*n* = 5).

Biosamples	Spiked concentration (ng/mL)	Intraday			Interday		
		Calculated concentration (ng/mL)	Precision (RSD, %)	Accuracy (%)	Calculated concentration (ng/mL)	Precision (RSD, %)	Accuracy (%)
LVFX in liver tissue	10.02	9.33 ± 1.03	11.03	93.07	9.18 ± 0.55	5.94	91.59
	100.20	104.61 ± 3.89	3.72	104.40	103.86 ± 4.09	3.94	103.65
	5010.00	4815.20 ± 158.68	3.30	96.11	4803.63 ± 111.96	2.33	95.88

LVFX in urine	10.02	9.49 ± 0.85	8.99	94.67	10.46 ± 0.51	4.85	104.35
	200.40	209.45 ± 10.49	5.01	104.52	207.55 ± 12.98	6.25	103.57
	4008.00	4183.48 ± 102.27	2.44	104.39	3875.60 ± 97.53	2.52	96.70

LVFX in feces	10.02	9.54 ± 0.86	9.03	95.17	10.54 ± 0.73	6.90	105.16
	20.04	20.56 ± 1.16	5.66	102.58	19.54 ± 0.91	4.64	97.53
	200.40	215.78 ± 13.68	6.34	107.67	190.05 ± 20.49	10.78	94.83

**Table 4:** Recovery and matrix effect of LVFX in rat liver tissues, urine, and feces (*n* = 5).

Biosamples	Spiked concentration (ng/mL)	Extraction recovery		Matrix effect	
		Mean ± SD	RSD (%)	Mean ± SD	RSD (%)
LVFX in liver tissue	10.02	90.84 ± 9.21	10.14	93.09 ± 6.72	7.22
	100.20	89.11 ± 6.24	7.00	103.03 ± 2.50	2.43
	5010.00	89.60 ± 4.69	5.24	97.64 ± 6.08	6.23

LVFX in urine	10.02	94.07 ± 7.88	8.38	93.02 ± 4.09	4.39
	200.40	91.84 ± 10.21	11.11	89.06 ± 6.53	7.33
	4008.00	93.12 ± 5.71	6.14	103.18 ± 9.78	9.48

LVFX in feces	10.02	93.94 ± 8.39	8.93	89.96 ± 4.86	5.41
	20.04	91.36 ± 7.60	8.31	93.00 ± 5.36	5.76
	200.40	101.13 ± 7.14	7.06	92.67 ± 9.76	10.53

**Table 5:** Stability of LVFX under different storage conditions (*n* = 5).

Biosamples	Spiked concentration (ng/mL)	Calculated concentration (ng/mL)		
		Room temperature	Cold storage	Three freeze-thaw cycles
LVFX in liver tissue	10.02	9.36 ± 0.44	9.28 ± 0.85	9.60 ± 0.94
	100.20	102.33 ± 8.31	102.92 ± 10.23	108.10 ± 3.75
	5010.00	4887.83 ± 105.12	4738.62 ± 157.74	4798.11 ± 159.01

LVFX in urine	10.02	8.98 ± 0.97	9.22 ± 0.99	10.50 ± 0.75
	200.40	207.47 ± 10.63	212.35 ± 11.46	197.20 ± 10.17
	4008.00	3869.70 ± 143.60	3888.71 ± 161.29	3833.85 ± 91.54

LVFX in feces	10.02	9.02 ± 0.84	9.74 ± 0.67	9.33 ± 0.82
	20.04	20.96 ± 1.66	18.75 ± 1.19	21.44 ± 0.83
	200.40	192.05 ± 17.68	191.49 ± 18.34	196.81 ± 13.40

**Table 6:** Dilution integrity of LVFX in urine and fecal samples (*n* = 5).

Biosamples	Mean ± SD	RSD (%)
LVFX in urine	965.35 ± 39.82	4.12
LVFX in feces	100.51 ± 9.00	8.96

## Data Availability

The data used to support the findings of this study are included within the article.

## References

[1] Lim J. W., Chee S. X., Wong W. J., He Q. L., Lau T. C. (2018). Traditional Chinese medicine: herb-drug interactions with aspirin. *Singapore Medical Journal*.

[2] Ling C.-Q., Fan J., Lin H.-S. (2018). Clinical practice guidelines for the treatment of primary liver cancer with integrative traditional Chinese and Western medicine. *Journal of Integrative Medicine*.

[3] Wang W. J., Zhang T. (2017). Integration of traditional Chinese medicine and Western medicine in the era of precision medicine. *Journal of Integrative Medicine*.

[4] Scheife R. T., Hines L. E., Boyce R. D. (2015). Consensus recommendations for systematic evaluation of drug–drug interaction evidence for clinical decision support. *Drug Safety*.

[5] Vora A., Varghese A., Kachwala Y. (2019). Eugenia jambolana extract reduces the systemic exposure of Sitagliptin and improves conditions associated with diabetes: a pharmacokinetic and a pharmacodynamic herb-drug interaction study. *Journal of Traditional and Complementary Medicine*.

[6] Blonk B., Cock I. E. (2019). Interactive antimicrobial and toxicity profiles of Pittosporum angustifolium Lodd. extracts with conventional antimicrobials. *Journal of Integrative Medicine*.

[7] Chen X. W., Serag E. S., Sneed K. B. (2011). Clinical herbal interactions with conventional drugs: from molecules to maladies. *Current Medicinal Chemistry*.

[8] Oliphant C. M., Green G. M. (2002). Quinolones: a comprehensive review. *American Family Physician*.

[9] Zhao X., Wu J.-F., Xiu Q.-Y. (2014). A randomized controlled clinical trial of levofloxacin 750 mg versus 500 mg intravenous infusion in the treatment of community-acquired pneumonia. *Diagnostic Microbiology & Infectious Disease*.

[10] Poole M., Anon J., Paglia M., Xiang J., Kahn J. (2006). A trial of high-dose, short-course levofloxacin for the treatment of acute bacterial sinusitis. *Otolaryngology–Head and Neck Surgery*.

[11] Lin H. A., Yang Y. S., Wang J. X. (2016). Comparison of the effectiveness and antibiotic cost among ceftriaxone, ertapenem, and levofloxacin in treatment of community-acquired complicated urinary tract infections. *Journal of Microbiology, Immunology and Infection*.

[12] Cui H. (2016). Analysis of adverse reaction of levofloxacin and clinical rational drug use. *Journal of Mathematical Medicine*.

[13] Wu H. (2001). Analysis of 255 levofloxacin ADR reports. *Chinese Journal of Pharmacoepidemiology*.

[14] Shan A., Zhao G., Qian S. (2012). Investigation and analysis the adverse drug reaction of levofloxacin on domestic and foreign case report. *The Chinese Journal of Clinical Pharmacology*.

[15] Zheng L., Lu Y., Cao X. (2014). Evaluation of the impact of *Polygonum capitatum*, a traditional Chinese herbal medicine, on rat hepatic cytochrome P450 enzymes by using a cocktail of probe drugs. *Journal of Ethnopharmacology*.

[16] Liao S.-G., Li-Juan Z., Fa S. (2011). Antibacterial and anti-inflammatory effects of extracts and fractions from *Polygonum capitatum*. *Journal of Ethnopharmacology*.

[17] Ministry of Health of the People’s Republic of China (2015). *Pharmacopoeia of the People’s Republic of China*.

[18] Yide C., Yongke Z., Qi L., Shaokang S. (2018). Evaluation of clinical effect and therapeuticsafety of Relinqing combined with Levofloxacin in the treatment of chronic bacterial prostatitis. *China Modern Medicine*.

[19] Yinkui S. (2014). Relinqing granules combined with levofloxacin for treating 56 cases of urinary tract infection. *China Pharmaceuticals*.

[20] Lu X. (2019). Clinical efficacy of relinqing granule combined with levofloxacin mesylate in the treatment of urinary tract infection. *Chinese Journal of Clinical Rational Drug Use*.

[21] Roy B., Das A., Bhaumik U. (2010). Determination of gemifloxacin in different tissues of rat after oral dosing of gemifloxacin mesylate by LC–MS/MS and its application in drug tissue distribution study. *Journal of Pharmaceutical & Biomedical Analysis*.

[22] Ma F.-W., Deng Q.-F., Zhou X. (2016). The tissue distribution and urinary excretion study of gallic acid and protocatechuic acid after oral administration of *Polygonum capitatum* extract in rats. *Molecules*.

[23] Li Q., Xie L., Zhang J., Weina P. J. (2008). The distribution pattern of intravenous [14C] artesunate in rat tissues by quantitative whole-body autoradiography and tissue dissection techniques. *Journal of Pharmaceutical and Biomedical Analysis*.

[24] Lu Y., Gong Z., Xie Y. (2016). Herb-drug interaction: effects of relinqing granule on the pharmacokinetics of ciprofloxacin, sulfamethoxazole, and trimethoprim in rats. *Evidence-Based Complementary and Alternative Medicine*.

[25] Zheng L., Chen H., Sun H. (2019). Comparative study on tissue distribution of *Polygonum capitatum* extract between pyelonephritis model and normal rats. *Chinese Journal of New Drugs*.

[26] Zhang L., Li X., Wang Y., Liu M., Wang X., Niu L. (2019). Tissue distribution of total glucosides of paeony in normal rats. *Natural Product Research and Development*.

[27] Ma B. L., Ma Y. M. (2016). Pharmacokinetic herb-drug interactions with traditional Chinese medicine: progress, causes of conflicting results and suggestions for future research. *Drug Metabolism Reviews*.

[28] Bo L., Zhao B., Yang L. (2016). Herb-drug enzyme-mediated interactions and the associated experimental methods: a review. *Journal of Traditional Chinese Medicine*.

[29] Liu Z. J., Jin Q. I., Zhu D. N., Bo-Yang Y. U. (2008). Chemical constituents from *Polygonum capitatum* and their antioxidation activities in vitro. *Journal of Chinese Medicinal Materials*.

[30] Yongjun L., Hongfeng L., Yonglin W., Wanyun S., Changqi H. (2000). Studies on the chemical constituents of flavonoids from *Polygonum capitatum*. *Journal of Chinese Pharmaceutical Sciences*.

[31] Chen Z., Zheng S., Li L., Jiang H. (2014). Metabolism of flavonoids in human: a comprehensive review. *Current Drug Metabolism*.

[32] Ito T., Yano I., Tanaka K., Inui K. I. (1997). Transport of quinolone antibacterial drugs by human P-glycoprotein expressed in a kidney epithelial cell line, LLC-PK1. *Journal of Pharmacology and Experimental Therapeutics*.

[33] Beydokthi S. S., Sendker J., Brandt S., Hensel A. (2017). Traditionally used medicinal plants against uncomplicated urinary tract infections: hexadecyl coumaric acid ester from the rhizomes of *Agropyron repens* (L.) P. Beauv. with antiadhesive activity against uropathogenic *E. coli*. *Fitoterapia*.

[34] Du C., Ni J., Zhang L. (2020). Effect of qinglin granule assisted levofloxacin on the removal of bacteria and inflammatory factors in urine of patients with urinary tract infection. *Anti Infective Pharmaceutical Journal*.

